# Nigerian Results-Based Financing Fellowship: A Strategic Approach for Sustaining Results-Based Financing in Nigeria

**DOI:** 10.3389/fpubh.2020.593175

**Published:** 2020-12-11

**Authors:** Olusola Olabisi Ogunseye

**Affiliations:** Department of Environmental Health Sciences, Faculty of Public Health, College of Medicine, University of Ibadan, Ibadan, Nigeria

**Keywords:** results-based financing, input-based financing, technical assistance, primary health care, health system, reform, Nigeria

## Introduction

There is an emergency in the Nigerian primary health care (PHC) system. Majority of PHC facilities are in a sordid and lugubrious state (1). Despite substantial government spending and policy initiatives toward revitalizing the PHC system, it remains largely deplorable across the World Health Organization (WHO) health system building blocks (2). To this end, the government of Nigeria in collaboration with the World Bank launched a Results-Based Financing (RBF) project under the Project Name: Nigeria State Health Investment Project (NSHIP) with the goal of improving service utilization and quality of healthcare services in Nigeria (2, 3). Due lack of in-country capacity, Oxford Policy Management (OPM) was contracted by National Primary Health Care Development Agency (NPHCDA) in 2014 to provide technical assistance to NSHIP for the implementation of RBF in Nigeria. In order to bridge the capacity gap at the exit of OPM in 2018, Nigerian Results-Based Financing Fellowship was conceptualized jointly by OPM and NPHCDA to train 14 Nigerians to a level whereby they are able to take on technical assistance roles in NPHCDA for continued implementation and possible scale-up of RBF in Nigeria (4). This paper describes the 10-months RBF fellowship, the competencies acquired and its usefulness as strategic approach for the sustainability of RBF in Nigeria.

## Description of RBF Fellowship

The RBF fellowship was conceptualized to train 14 Nigerians over a period of 10 months on technical (health systems, health financing, RBF design, and RBF implementation), professional (project management, presenting scientific data, and managing for efficiency and effectiveness) and behavioral (integrity and collaborative work) competencies through an approach that involved formal learning blocks, supervised and assessed practice, and internship, to ensure the Fellows become professionals capable of continuing and possibly expand RBF in Nigeria's quest to achieve universal health coverage (4, 5). During formal learning blocks, modules were delivered by world-class experts and Results-Based Financing Technical Assistants (RBF-TAs) while Fellows were under the tutelage and mentoring of RBF-TAs during the supervised and assessed practice.

## Formal Learning Blocks

Formal learning blocks adopted in-person classroom teaching model where relevant topics were delivered in form of modules. These modules were designed to help Fellows become well-rounded leaders and managers in the Nigerian health system with integrity, efficiency, and effectiveness. The formal learning blocks targeted the 3-fold competency areas of the fellowship; technical, professional and behavioral competencies.

## Supervised and Assessed Practice

Supervised and assessed practice was an avenue for Fellows to apply theoretical knowledge acquired during formal learning blocks in selected pilot RBF implementing States in Nigeria. It provided a platform for Fellows to undertake managerial and administrative roles, involving practical assignments in a routine RBF setting.

## Personal Development Plan and Reflective Learning Log

The Personal Development Plan (PDP) is an interactive, easy-to-read and comprehensive document designed by Fellows during the first formal learning block detailing goals, learning gaps and strategies to achieve objectives of the fellowship across the competency areas. The PDP is a working document that assesses Fellows' competence at baseline, track their progress, evaluate areas of improvements *vis-a-vis* the baseline, appraise capacity gaps and revise strategies for achieving them. Fellows reviewed and updated the PDP at least twice over the course of the fellowship.

The reflective learning log is a personal learning record produced through insightful thinking on what is learned and shows progress toward achieving learning objectives stated in the PDP. Fellows made entries in the log during the formal learning blocks, supervised and assessed practice, and internship. Basically, the PDP and reflective learning log are closely linked and they serve as powerful tools for assessing Fellows' learning trajectory and overall effectiveness of the fellowship.

## Work-Based Written Assignments

In addition to practical and field-based assignments during supervised and assessed practice and internship, Fellows were assessed based on three work-based written assignments over the course of the fellowship. Each work-based written assignment is an article of about 2,000 words on specific topics in health financing, RBF design and RBF implementation. These articles were also presented and critiqued by Fellows during State-based tutorial group meetings to enhance scholarship and critical reasoning skills of Fellows.

## Internship

Internship was a period for Fellows to undertake work-based practical assignments and apply the knowledge acquired from modules and syllabi in formal learning blocks and previous supervised and assessed practice. Fellows performed the roles and responsibilities of RBF-TA during internship with minimal supervision. The period of internship offered great opportunities for hands-on, work-based practical experience. The internship rounded off the transformational process required for Fellows in the 3-fold competency areas targeted by the fellowship.

## Discussion

### RBF Fellowship as Strategic Approach

Even though Nigeria is not in short supply of health policies, the country has always lacked cutting-edge resolve to fully implement those policies. Evidences suggest that the currently implemented input-based financing mechanism has failed to yield desired results with the country's health system performing poorly in critical maternal and child health outcomes and lagging behind other countries (6–9). While evidences of RBF impact in Nigeria are scanty, there are few studies that indicate RBF offers improvement in access, utilization and quality of health services (7, 9–13). RBF possesses great potential and could serve as a tool to reform the Nigerian health system, particularly primary health care, hence, sustaining the gains of RBF in Nigeria will rely substantially on institutionalizing RBF fellowship as a unique platform for continuous recruitment and training of health professionals with competencies required to drive primary health care policies for the attainment of universal health coverage.

## Conclusion

This paper documents the 10-months RBF fellowship in light of its objectives, framework, and targeted competencies while emphasizing the need for its utilization as strategic approach for the sustenance and expansion of RBF in Nigeria. The RBF fellowship was an impactful, highly enriching, result-driven, competency-targeted programme. This programme will reinforce RBF approach in Nigeria, which in turn could set the tone for Nigeria's progress toward universal health coverage on adoption as a tool for national policy in healthcare.

## Author Contributions

OOO conceptualized, documented, wrote, and finalized the manuscript.

## Conflict of Interest

The author declares that the research was conducted in the absence of any commercial or financial relationships that could be construed as a potential conflict of interest.
